# Resolutions of Respect

**Published:** 1892-12

**Authors:** W. S. Kooker, Zeno S. Keil, J. Rein Keelor


### RESOLUTIONS OF RESPECT.

*Resolved*, That in the sudden and unlooked-for death of our late fellow-member, Dr. J. B. Fretz, the Pennsylvania State Veterinary Medical Association has lost one, who, since the origin of our Association, had its interests at heart, and by his counsel, courteous manner, gentlemanly conduct, and many good traits has endeared himself to all, and that we will ever hold his memory dear.

*Resolved*, That these resolutions be spread on the minutes of the Association, and a copy be sent to his family.

(Signed) W. S. KOOKER, V.S.,
ZENO S. KEIL, V.S.,
J. REIN, KEELOR, V.S.

